# Surprising absence of strong homonuclear coupling at low magnetic field explored by two-field nuclear magnetic resonance spectroscopy

**DOI:** 10.5194/mr-1-237-2020

**Published:** 2020-10-14

**Authors:** Ivan V. Zhukov, Alexey S. Kiryutin, Ziqing Wang, Milan Zachrdla, Alexandra V. Yurkovskaya, Konstantin L. Ivanov, Fabien Ferrage

**Affiliations:** 1 International Tomography Center, Siberian Branch of the Russian Academy of Sciences, 630090 Novosibirsk, Russia; 2 Department of Physics, Novosibirsk State University, 630090 Novosibirsk, Russia; 3 Laboratoire des Biomolécules (LBM), Département de chimie, École normale supérieure, PSL University, Sorbonne Université, CNRS, 75005 Paris, France

## Abstract

Strong coupling of nuclear spins, which is achieved when their scalar coupling 2πJ is greater than or comparable to the difference Δω in their Larmor precession frequencies in an external magnetic field, gives rise to efficient coherent longitudinal polarization transfer. The strong coupling regime can be achieved when the external magnetic field is sufficiently low, as Δω is reduced proportional to the field strength. In the present work, however, we demonstrate that in heteronuclear spin systems these simple arguments may not hold, since heteronuclear spin–spin interactions alter the Δω value. The experimental method that we use is two-field nuclear magnetic resonance (NMR), exploiting sample shuttling between the high field, at which NMR spectra are acquired, and the low field, where strong couplings are expected and at which NMR pulses can be applied to affect the spin dynamics. By using this technique, we generate zero-quantum spin coherences by means of a nonadiabatic passage through a level anticrossing and study their evolution at the low field. Such zero-quantum coherences mediate the polarization transfer under strong coupling conditions. Experiments performed with a ${}^{13}C$ -labeled amino acid clearly show that the coherent polarization transfer at the low field is pronounced in the ${}^{13}C$ spin subsystem under proton decoupling. However, in the absence of proton decoupling, polarization transfer by coherent processes is dramatically reduced, demonstrating that heteronuclear spin–spin interactions suppress the strong coupling regime, even when the external field is low. A theoretical model is presented, which can model the reported experimental results.

## 1 Introduction

The topological and conformational information provided by scalar couplings lies at the foundation of the analytical power of nuclear magnetic resonance (NMR) spectroscopy (Ernst et al., 1987; Keeler, 2005; Levitt, 2008; Cavanagh, 2007). The strong coupling case is encountered when scalar coupling constants are not negligible with respect to the difference of resonance frequency between the coupled spins (Keeler, 2005). Understanding strong scalar couplings and their spectral signature was essential when NMR was introduced for chemical analysis, which was typically performed at magnetic fields considered today as low (Bodenhausen et al., 1977; Pfändler and Bodenhausen, 1987). Modern high-field NMR is widely based on the exploitation of weak scalar couplings, so that strong scalar couplings have remained a nuisance, in particular in aromatic spin systems (Vallurupalli et al., 2007; Foroozandeh et al., 2014). Recently, the development and availability of benchtop NMR spectrometers operating at low or moderate magnetic fields (Grootveld et al., 2019) has revived the interest in the understanding of strong scalar couplings in conventional NMR.

Contrary to conventional NMR, NMR at near-zero or ultralow magnetic fields (ZULF–NMR) explores the benefits of NMR in the strong scalar-coupling regime. At such magnetic fields, typically smaller than 1 µT , scalar-coupling interactions dominate all Zeeman interaction and dictate the eigenstates of spin systems and transition energies obtained in spectra (Ledbetter et al., 2011; Tayler et al., 2017; Blanchard and Budker, 2016). However, for homonuclear couplings, the transition between the weak and strong coupling regimes occurs in a range of magnetic fields where the Zeeman interaction is still dominant (Ivanov et al., 2006, 2008, 2014; Appelt et al., 2010; Türschmann et al., 2014). This transition between weak and strong couplings can be investigated by varying the magnetic field applied to the sample on a high-field magnet, which is usually performed by moving the sample through the stray field with a shuttle system (Roberts and Redfield, 2004a, b; Redfield, 2012; Wagner et al., 1999; Bryant and Korb, 2005; Goddard et al., 2007; Chou et al., 2016, 2017; Charlier et al., 2013; Cousin et al., 2016a, b; Zhukov et al., 2018; Kiryutin et al., 2016). These studies have highlighted the effects of level anticrossings (LACs; Miesel et al., 2006; Ivanov et al., 2014). When the passage through a LAC is slow, the transition is adiabatic, and the population of eigenstates is smoothly converted to the new eigenstates. When the transition is fast, coherences can be generated between the new eigenstates and time oscillations of the population of high-field eigenstates can be observed (Pravdivtsev et al., 2013; Kiryutin et al., 2013). As usual, nonadiabatic variation, which gives rise to excitation of coherences, means that the adiabatic eigenstates of the spin system change with time fast compared to the rate of internal evolution of the system. Specifically, for each pair of adiabatic states, |i〉 and |j〉 , the following parameter:

$$\xi_{ij}=\frac{\langle j\left|\frac{d}{dt}\right|i\rangle }{\omega_{ij}}$$

is much greater than one (here $\omega_{ij}$ is the energy difference between the states measured in angular frequency units). When $\xi_{ij}\ll 1$ , switching is adiabatic and populations follow the time-dependent eigenstates. This phenomenon has been observed on a variety of homonuclear spin systems. Heteronuclear scalar couplings have been shown to alter LACs in homonuclear spin systems (Korchak et al., 2012); yet, the properties of such heteronuclear couplings on LACs are not fully understood – particularly in spin systems with extensive networks of homo- and heteronuclear scalar couplings.

Here, we investigate the effect of heteronuclear scalar couplings on LACs in a spin system typical of biomolecular NMR, a uniformly ${}^{13}C$ -labeled amino acid (leucine), which combines extensive networks of homo- and heteronuclear scalar couplings. Essentially, we exploit the ability to apply composite pulse decoupling on our two-field NMR spectrometer (Cousin et al., 2016a) to switch heteronuclear scalar couplings at low magnetic field on and off. We demonstrate that heteronuclear scalar couplings alter LACs by sustaining the weak coupling regime in a carbon-13 homonuclear spin system. Composite pulse decoupling at low magnetic field restores the strong scalar-coupling regime in the carbon-13 nuclei of the isopropyl group of leucine at 0.33 T. Our results identify how heteronuclear couplings alter homonuclear couplings at low magnetic fields, which could be exploited in low-field NMR methodology and may be considered in further developments of total correlation spectroscopy (TOCSY; Braunschweiler and Ernst, 1983) mixing sequences in high-field NMR.

## 2 Methods

### 2.1 Sample preparation

**Figure 1:**
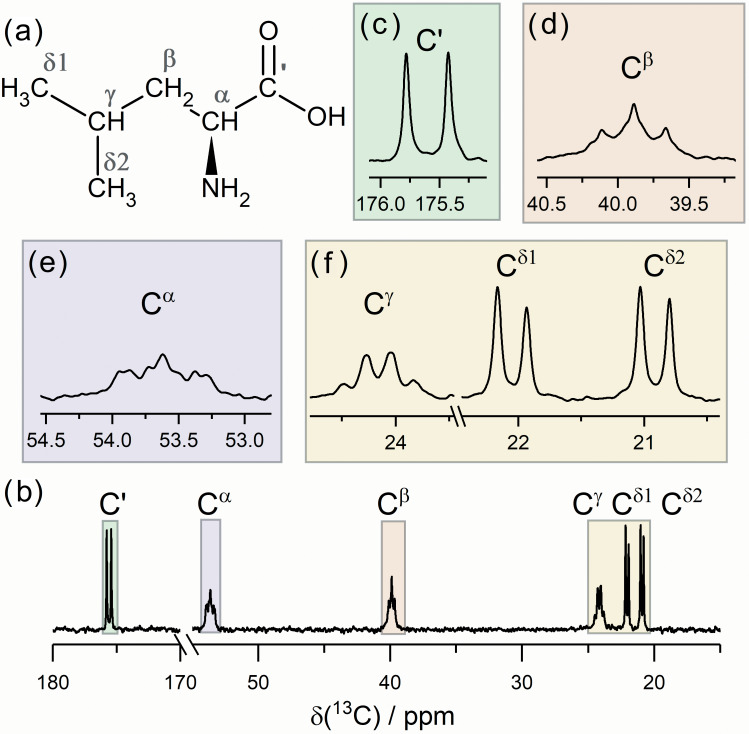
The structure of ${}^{13}C$ , ${}^{15}N$ L-leucine **(a)** and 150.9 MHz ${}^{13}C$ –NMR spectrum **(b)** under broadband ${}^{1}H$ decoupling. The signal of each carbon nucleus is also shown separately **(c–f)**. The multiplet structure in the spectrum is due to ${}^{13}C{-}^{13}C$ and ${}^{13}C{-}^{15}N$ scalar interactions.

Experiments have been performed using the following sample: 76 mM 99 % enriched ${}^{13}C$ , ${}^{15}N$ -labeled L-leucine (Leu) in 90 % $H_{2}O$ 10 % $D_{2}O$ solution. ${}^{13}C$ - and ${}^{15}N$ -enriched L-leucine were purchased from Sigma-Aldrich and used as it stands. The ${}^{13}C$ –NMR spectrum of the labeled Leu molecule is shown in Fig. 1. We also show, separately, the signals of the individual ${}^{13}C$ nuclei. Broadband proton decoupling was used to simplify the spectrum. Here, we will focus on a three-spin system formed by the $C^{\gamma }$ and two $C^{\delta }$ nuclei of the isopropyl moiety. We will study polarization transfer in this subsystem upon fast switching of the external magnetic field obtained by a transfer of the sample though the stray field of the high-field NMR magnet.

### 2.2 Field-cycling NMR experiments

NMR experiments were performed on a two-field NMR spectrometer (Cousin et al., 2016a) with fast sample shuttling (Charlier et al., 2013). The high-field $B_{\mathrm{HF}}=14.1$ T is the detection field of a 600 MHz NMR spectrometer, while the low field is $B_{\mathrm{LF}}=0.33$ T corresponding to 14 MHz ${}^{1}H$ Larmor frequency. The magnetic field in the low-field center is sufficiently homogeneous (inhomogeneities of the order of 10 parts per million – ppm) so that radiofrequency (RF) pulses can be applied by using a triple-resonance NMR probe, as described previously (Cousin et al., 2016a).

**Figure 2:**
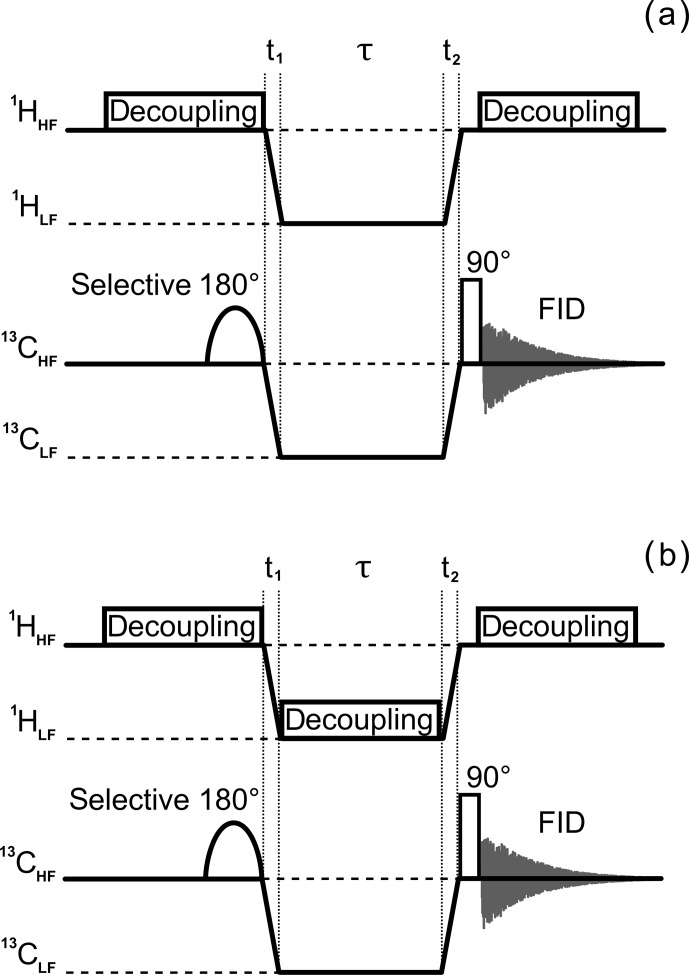
Experimental protocols of field-cycling NMR experiments without ${}^{1}H$ decoupling at the low field **(a)** and with 24 kHz WALTZ-64 ${}^{1}H$ decoupling at the low field **(b)**. Details of the experiments are as follows. A 6.2 kHz WALTZ-64 composite pulse decoupling on the proton channel was applied at $B_{\mathrm{HF}}$ during 100 ms prior to a selective 180 ${}^{\circ }$ pulse in order to enhance ${}^{13}C$ polarization by the nuclear Overhauser effect. The sample shuttle transfer times, $t_{1}$ and $t_{2}$ , were 107 and 94 ms, respectively. Selective inversion was performed with a RE-BURP pulse (Geen and Freeman, 1991), with a duration of 46.4 ms, at the $C^{\delta 2}$ resonant frequency covering ca. 100 Hz bandwidth. The delay τ at the low field was incremented with a 1 ms step. After a sample transfer to the high field, a hard 90 ${}^{\circ }$ pulse-generated ${}^{13}C$ transverse magnetization free induction decay (FID) acquisition was done during 1.56 s under 6.2 kHz WALTZ-64 proton decoupling.

Field-cycling NMR experiments were run according to the pulse sequences depicted in Fig. 2. First, a nonequilibrium state is generated at $B_{\mathrm{HF}}$ by applying a selective π pulse to the $C^{\delta 2}$ nucleus; i.e., a shaped refocusing band-selective uniform-response pure-phase (RE-BURP) pulse (Geen and Freeman, 1991) for which the pulse duration was 46.4 ms, and the peak RF-field amplitude was adjusted to cover ca. 100 Hz bandwidth around the center of $C^{\delta 2}$ signal. The RE-BURP-shaped pulse was used since it is less sensitive to the initial nuclear magnetization state than inversion band-selective uniform-response pure-phase (I-BURP; Geen and Freeman, 1991) and has a narrow excitation profile. To improve the selectivity of the pulse, simultaneous proton decoupling was used, which reduces multiplet overlap in the ${}^{13}C$ NMR spectrum. Following this preparation, the sample was shuttled from the high-field center to the low-field center $B_{\mathrm{HF}}\to B_{\mathrm{LF}}$ with a duration $t_{1}=107$ ms. The field jump is fast enough to be nonadiabatic, and it is aimed at exciting a spin coherence. Subsequently, the coherence evolves at $B_{\mathrm{LF}}$ during a variable time period τ . The shuttle transfer back to the high-field center leads to a second field jump $B_{\mathrm{LF}}\to B_{\mathrm{HF}}$ with a duration of $t_{2}=94$ ms. This second nonadiabatic field jump to $B_{\mathrm{HF}}$ converts the coherence into a population difference. Detection is performed after a π/2 pulse on the carbon-13 channel in the presence of proton decoupling. We perform two types of experiments in which the carbon spin coherence (zero-quantum coherence – ZQC) evolves at $B_{\mathrm{LF}}$ in the absence (see Fig. 2a) and in the presence (see Fig. 2b) of proton composite pulse decoupling. Decoupling at $B_{\mathrm{LF}}$ has been performed using a composite pulse decoupling pulse together with the wideband alternating-phase low-power technique for zero-residual splitting (WALTZ) decoupling with MLEV-64 supercycle (Shaka et al., 1983; Levitt et al., 1982) at the low field on the proton RF channel (operating at 14 MHz, corresponding to the proton NMR frequency at 0.33 T). Composite pulse decoupling is used because of the rather high field inhomogeneity at 0.33 T, which is of the order of 10 ppm; under such conditions, continuous-wave decoupling would require more power, potentially giving rise to sample heating. The τ dependence of polarization is expected to be oscillatory due to the coherent polarization exchange within the expectedly strongly coupled system of the $C^{\gamma }$ and two $C^{\delta }$ carbon-13 nuclei.

## 3 Theory

### 3.1 Polarization transfer in a three-spin system

In this subsection, we provide a theoretical description of the field-cycling NMR experiments. First, we present the analytical treatment of polarization transfer among two nuclei of the same kind, here spin $I_{1}$ and spin $I_{2}$ (e.g., two carbon-13 nuclei), in the presence of a third spin S , which can be a heteronucleus (e.g., here a proton). This is the minimal system allowing us to detail the effect of a heteronucleus on polarization transfer among strongly coupled spins. We assume that spins $I_{1}$ and $I_{2}$ are in strong coupling conditions, meaning that the difference, Δω , in their Zeeman interaction frequencies with the external field is smaller than or comparable to the scalar coupling constant, $2\pi J_{12}$ , between them. When the strong coupling regime is achieved, the zero-quantum part of the scalar coupling, given by the operator $\left\{{\hat{I}}_{1+}{\hat{I}}_{2-}+{\hat{I}}_{1-}{\hat{I}}_{2+}\right\}$ , becomes active, giving rise to flips and flops of spins $I_{1}$ and $I_{2}$ . The couplings to the third spin S , namely $J_{13}$ and $J_{23}$ , are assumed to be unequal (otherwise coupling to the proton gives rise to an identical shift of the NMR frequencies of spins 1 and 2 and does not modify the eigenstates of this subsystem). The Hamiltonian of the spin system can be written as follows (in 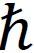 units):

1 $$\begin{array}{rl} {\hat{H}}_{\mathrm{CCH}}= & \;-\omega_{1}{\hat{I}}_{1z}-\omega_{2}{\hat{I}}_{2z}-\omega_{3}{\hat{S}}_{z}+2\pi J_{12}\left({\hat{I}}_{1}\cdot {\hat{I}}_{2}\right)\\ & +2\pi J_{13}{\hat{I}}_{1z}{\hat{S}}_{z}+2\pi J_{23}{\hat{I}}_{2z}{\hat{S}}_{z}. \end{array}$$

Here ${\hat{I}}_{1}$ , ${\hat{I}}_{2}$ and $\hat{S}$ are the spin operators; $\omega_{1}=\gamma_{I}\left(1+\delta_{1}\right)B$ , $\omega_{2}=\gamma_{I}\left(1+\delta_{2}\right)B$ and $\omega_{3}=\gamma_{S}\left(1+\delta_{3}\right)B$ stand for the NMR frequencies of the corresponding nuclei (with $\gamma_{I,\;S}$ being the corresponding gyromagnetic ratios and $\delta_{i}$ being the chemical shifts). We assume that the heteronucleus S is coupled weakly to I spins due to the large difference in their NMR frequencies, i.e., $\left|\omega_{1}-\omega_{3}\right|,\left|\omega_{2}-\omega_{3}\right|\gg \left|\omega_{1}-\omega_{2}\right|,2\pi J_{13},2\pi J_{23}$ , and keep only the secular part of the heteronuclear coupling Hamiltonian.

In the present case, the nuclear magnetic number, $m_{S}$ , of spin S is a good quantum number, which is conserved because ${\hat{S}}_{z}$ commutes with the Hamiltonian. For this reason, it is possible to find the solution for the spin dynamics of spins $I_{1}$ and $I_{2}$ for two separate cases, which corresponds to the two different values of $m_{S}$ being $+\frac{1}{2}$ and $-\frac{1}{2}$ , i.e., spin S is in the spin-up $\left|\alpha \rangle \right.$ state or spin-down $\left|\beta \rangle \right.$ state. In each case, the Hamiltonian of the carbon subsystem is as follows:

2 $$\begin{array}{rl} {\hat{H}}_{\mathrm{CC}}= & \;-\left\{\omega_{1}-2\pi J_{13}S_{z}\right\}{\hat{I}}_{1z}-\left\{\omega_{2}-2\pi J_{23}S_{z}\right\}{\hat{I}}_{2z}\\ & +2\pi J_{12}\left({\hat{I}}_{1}\cdot {\hat{I}}_{2}\right). \end{array}$$

Hence, in the Hamiltonian given by Eq. (1) we replace the ${\hat{S}}_{z}$ operator by the $m_{S}$ value, which is $\pm \frac{1}{2}$ . Hence, the Δω value is modified, and it depends on the $m_{S}$ value, as follows:

3 $$\Delta \omega_{\pm }=\left\{\omega_{1}-\omega_{2}\right\}\mp \pi \left\{J_{13}-J_{23}\right\}=\Delta \omega \mp \pi \cdot \Delta J.$$

The eigenstates of the subsystem of spin 1 and spin 2 are as follows:

4 $$\begin{array}{l} \left|1\rangle \right.=\left|\alpha \alpha \rangle \right.,{\left|2\rangle \right.}_{\pm }=\mathrm{cos}\theta_{\pm }\left|\alpha \beta \rangle \right.+\mathrm{sin}\theta_{\pm }\left|\beta \alpha \rangle \right.\\ {\left|3\rangle \right.}_{\pm }=-\mathrm{sin}\theta_{\pm }\left|\alpha \beta \rangle \right.+\mathrm{cos}\theta_{\pm }\left|\beta \alpha \rangle \right.,\left|4\rangle \right.=\left|\beta \beta \rangle \right.. \end{array}$$

Here, the mixing angle is given by the values of $\Delta \omega_{\pm }$ and $J_{12}$ : $\mathrm{tan}2\theta_{\pm }=2\pi J_{12}/\Delta \omega_{\pm }$ . When $\Delta \omega_{\pm }$ approaches zero, the mixing angle goes to $\frac{\pi }{4}$ , meaning that the eigenstates become singlet and triplet states; thus, the spins are strongly coupled. When $\Delta \omega_{\pm }$ is much greater than the coupling, the eigenstates are obviously the Zeeman states.

Even in this simple system, it is clear that the condition $\left|\omega_{1}-\omega_{2}\right|\ll 2\pi J_{12}$ is not sufficient to guarantee strong coupling of the two carbons. Indeed, when ΔJ is greater than Δω and $2\pi J_{12}$ , the carbon spins become weakly coupled in the two subensembles, corresponding to $m_{S}=\pm \frac{1}{2}$ .

How do heteronuclear couplings affect polarization transfer in the carbon system? We assume that at t=0 one of the spins has polarization ( $\langle I_{1z}\rangle =P_{0}$ ) and the other spin is not polarized ( $\langle I_{2z}\rangle =0$ ). Hereafter, it is convenient to use normalization $P_{0}=1$ . The state of the spin system is then given by the density operator as follows:

5 $$\sigma_{0}={\hat{I}}_{1z}.$$

As shown previously (Ivanov et al., 2006), in the two-spin system of $I_{1}$ and $I_{2}$ , in the absence of coupling to any other spin the polarization evolves with time as follows:

$$\langle {\hat{I}}_{1z}\rangle (t)=1-{\mathrm{sin}}^{2}\theta \frac{1-\mathrm{cos}\left[\omega_{\mathrm{ZQC}}t\right]}{2},$$

6 $$\langle {\hat{I}}_{2z}\rangle (t)={\mathrm{sin}}^{2}\theta \frac{1-\mathrm{cos}\left[\omega_{\mathrm{ZQC}}t\right]}{2},$$

where $\mathrm{tan}2\theta =2\pi J_{12}/\Delta \omega$ , and the oscillation frequency $\omega_{\mathrm{ZQC}}=\sqrt{\Delta \omega^{2}+{\left(2\pi J_{12}\right)}^{2}}$ is the frequency of the ZQC between the eigenstates |2〉 and |3〉 . Hence, coherent exchange in polarization is taking place. As Δω becomes smaller, the frequency of the oscillations decreases but the amplitude increases; at Δω→0 we obtain $\omega_{\mathrm{ZQC}}=2\pi \left|J_{12}\right|$ , and a complete exchange is possible when $t=1/\left(2J_{12}\right)$ .

**Figure 3:**
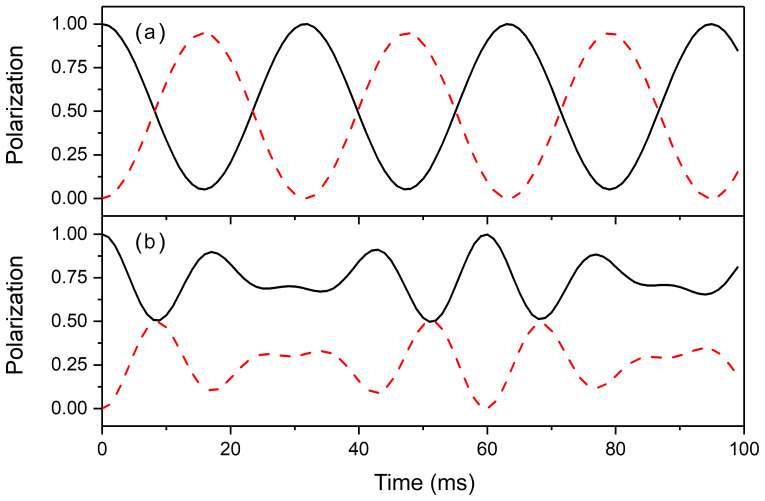
Polarization transfer between two strongly coupled nuclei **(a)** in the absence and **(b)** in the presence of a heteronucleus. Here, we present the time dependence of $\langle I_{1z}\rangle$ (black solid lines) and $\langle I_{2z}\rangle$ (red dashed lines) normalized to the initial value of $\langle I_{1z}\rangle$ . The density operator at time t=0 is $\sigma_{0}={\hat{I}}_{1z}$ . The parameters of the simulation were Δω/2π=10 Hz and $J_{12}=30$ Hz and **(a)** ΔJ=0 Hz and **(b)** ΔJ=100 Hz.

In the presence of scalar couplings to the third spin S , here a proton ( $I_{1}$ and $I_{2}$ are carbon-13 nuclei), the expressions should be modified; the evolution should be calculated for each specific spin state of the proton, namely 〈α〉 and 〈β〉 , and the sum of the two curves should be taken. We obtain this from the following expression:

$$\langle {\hat{I}}_{1z}\rangle (t)=1-{\mathrm{sin}}^{2}\theta_{+}\frac{1-\mathrm{cos}\left[\omega_{\mathrm{ZQC}}^{+}t\right]}{4}-{\mathrm{sin}}^{2}\theta_{-}\frac{1-\mathrm{cos}\left[\omega_{\mathrm{ZQC}}^{-}t\right]}{4},$$

7 $$\langle {\hat{I}}_{2z}\rangle (t)={\mathrm{sin}}^{2}\theta_{+}\frac{1-\mathrm{cos}\left[\omega_{\mathrm{ZQC}}^{+}t\right]}{4}+{\mathrm{sin}}^{2}\theta_{-}\frac{1-\mathrm{cos}\left[\omega_{\mathrm{ZQC}}^{-}t\right]}{4},$$

where the evolution frequencies are equal to $\omega_{\mathrm{ZQC}}^{\pm }=\sqrt{\Delta \omega_{\pm }^{2}+{\left(2\pi J_{12}\right)}^{2}}$ .

The time dependence of the expectation value for the longitudinal polarizations of spins $I_{1}$ and $I_{2}$ is presented in Fig. 3 in the presence and the absence of scalar couplings to a heteronucleus. In the absence of heteronuclear coupling, the two strongly coupled spins (the strong-coupling condition is fulfilled since $2\pi J_{12}>\Delta \omega$ ) almost completely exchange polarizations. The polarization transfer is of a coherent nature, and the frequency of the oscillations is close to the scalar coupling constant $J_{12}$ . In the presence of different heteronuclear scalar couplings to the third spin S , the time evolution changes considerably. The two spins are no longer in the regime of strong coupling, since $\left|\Delta \omega_{\pm }\right|>2\pi J_{12}$ . The efficiency of polarization transfer is reduced, and complete exchange of polarization is no longer possible. The time dependence also becomes more complex. Instead of a single frequency $\omega_{\mathrm{ZQC}}$ found in the previous case, here two frequencies appear, namely $\omega_{\mathrm{ZQC}}^{+}$ and $\omega_{\mathrm{ZQC}}^{-}$ . Hence, when couplings to heteronuclei are present, the condition $\Delta \omega ∼2\pi J_{12}$ does not guarantee that the homonuclei are in the strong coupling regime.

**Figure 4:**
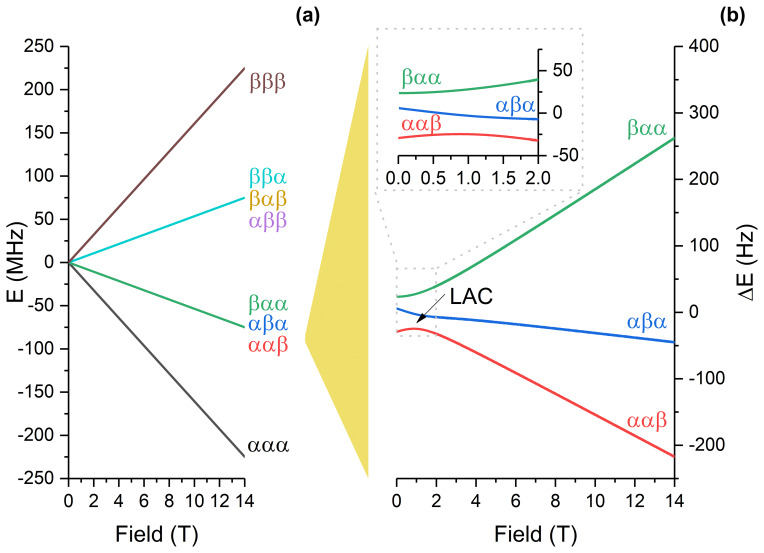
**(a)** Energy levels of the $\left\{C^{\gamma },C^{\delta 1},C^{\delta 2}\right\}$ spin system at variable magnetic field strength in the absence of scalar coupling with protons. Levels are assigned at the high field, where the spin system is weakly coupled. **(b)** Energy levels, corresponding to the ααβ and αβα states at the high field, have a level anticrossing (LAC) at 1.1 T, which is responsible for generation of the zero-quantum coherences. To visualize the energy levels better, in **(b)** we subtracted the large Zeeman energy from the actual energy to show the energy difference. The calculation is done using the parameters listed in Table 1 and neglecting carbon–proton couplings.

These results show that the interaction with a heteronucleus clearly alters polarization transfer in strongly coupled networks. Consequently, we expect strong effects of heteronuclear interactions on polarization transfers in systems with several heteronuclei. Notably, we anticipate that polarization transfer among strongly coupled carbon spins will be dramatically different in the presence of proton decoupling, which effectively removes proton–carbon spin–spin interactions.

### 3.2 Spin dynamics simulations

In addition to this simple model, we carried out numerical simulations in a realistic multispin system, i.e., the isopropyl group of carbon-13-labeled leucine. This spin system contains three ${}^{13}C$ nuclei $I_{1}$ , $I_{2}$ and $I_{3}$ , i.e., the $C^{\gamma }$ carbon-13 and the two $C^{\delta }$ carbon-13 nuclei. In addition, the spin system includes seven protons $S_{i}$ ; each $C^{\delta }$ nucleus is coupled to the three protons of the methyl group, and the $C^{\gamma }$ carbon-13 nucleus is coupled to one proton. We model the effects of fast field variation and coherent spin dynamics at the low field. We consider two cases, namely polarization transfer in the presence of and in the absence of proton decoupling.

The simulation method is as follows. The band-selective inversion pulse on spin $I_{3}$ generates the initial density operator for the three-spin I system as follows:

8 $$\sigma_{0}=\sigma (t=0)={\hat{I}}_{1z}+{\hat{I}}_{2z}-{\hat{I}}_{3z}.$$

Hence, we generate a population difference for the states |ααβ〉 , |αβα〉 and |βαα〉 . The first state is overpopulated, while the other two states are underpopulated. The three-spin system under study, namely $C^{\gamma }$ , $C^{\delta 1}$ and $C^{\delta 2}$ , has a LAC at $B=B_{\mathrm{LAC}}\approx 1.1$ T (see Fig. 4). Upon passage through a LAC during the field jump $B_{\mathrm{HF}}\to B_{\mathrm{LF}}$ due to the sample shuttle transfer, the population difference is expected to be converted into a coherence between the states which have the LAC; these adiabatic states correspond to the |ααβ〉 and |αβα〉 states at high fields. To calculate the actual spin state at $B=B_{\mathrm{LF}}$ , we solve numerically the Liouville–von Neumann equation for the spin density operator as follows:

9 $$\frac{d}{dt}\sigma =-i\left[\hat{H}(t),\sigma \right].$$

The Hamiltonian of the spin system at a magnetic field B is as follows:

10 $$\begin{array}{rl} \hat{H}(B) & =-\gamma_{C}B\sum_{i=1}^{3}\left(1+\delta_{Ci}\right){\hat{I}}_{iz}-\gamma_{H}B\sum_{j=1}^{7}\left(1+\delta_{Hj}\right){\hat{S}}_{iz}\\ & +2\pi \sum_{i\neq k}J_{Cik}\left({\hat{I}}_{i}\cdot {\hat{I}}_{k}\right)+2\pi \sum_{j\neq m}J_{Hjm}\left({\hat{S}}_{j}\cdot {\hat{S}}_{m}\right)\\ & +2\pi \sum_{i=1}^{3}\sum_{j=1}^{7}J{{}^{\prime }}_{ij}{\hat{I}}_{iz}{\hat{S}}_{jz}. \end{array}$$

Here $\gamma_{C}$ and $\gamma_{H}$ are the carbon and proton gyromagnetic ratios, $\delta_{Ci}$ and $\delta_{Hj}$ are the chemical shifts of the i th carbon and j th proton, $J_{Cik}$ is the scalar-coupling constant between the i th and k th carbon, $J_{Hjm}$ is the scalar-coupling constant between the j th and m th proton, $J{{}^{\prime }}_{ij}$ is the scalar-coupling constant between the i th carbon and j th proton, and ${\hat{I}}_{i}$ and ${\hat{S}}_{j}$ are the spin operator of the i th carbon and j th proton. Given the range of magnetic fields considered here, heteronuclear scalar couplings are considered to be weak.

**Table 1:** Parameters used for energy calculations. Proton–carbon direct scalar-coupling constants marked with an asterisk have been used in numerical simulation polarization transfers. Note: ppm – parts per million.

Chemical shifts	
$C^{\gamma }$	24.14 ppm
$C^{\delta 1}$	22.05 ppm
$C^{\delta 2}$	20.92 ppm
Scalar couplings	
$J(C^{\gamma }-C^{\delta 1})$	35 Hz
$J(C^{\gamma }-C^{\delta 2})$	35.4 Hz
$J(C^{\delta 1}-C^{\delta 2})$	0 Hz
$J(C^{\gamma }-H^{\gamma })$ ${}^{*}$	127.4 Hz
$J(C^{\delta 1}-H^{\delta 1})$ ${}^{*}$	124.8 Hz
$J(C^{\delta 2}-H^{\delta 2})$ ${}^{*}$	124.8 Hz

The precise values of the calculation parameters are given in Table 1. Since the magnetic field B changes with time, the Hamiltonian $\hat{H}$ is also time dependent. In the calculation, we consider three carbons and seven protons (six protons of the two ${\mathrm{CH}}_{3}$ groups and the γ proton). Using this Hamiltonian, we evaluate the density operator after the first field jump, $\sigma (t=t_{1})$ . The Liouville–von Neumann equation is integrated using 1 ms time increments and assuming that for each step the Hamiltonian is constant, similar to simulations carried out for relaxation experiments (Bolik-Coulon et al., 2020). In the calculation, we ignore relaxation effects, since the dimensionality of the relaxation super operator is too big for the multispin system considered here, and our focus is on coherent effects.

At $B=B_{\mathrm{LF}}$ , the density operator evolves under a constant Hamiltonian. At the end of the evolution period it becomes the following:

11 $$\sigma \left(t_{1}+\tau \right)=\mathrm{exp}\left(-i\hat{H}\left(B_{\mathrm{LF}}\right)\tau \right)\sigma \left(t_{1}\right)\mathrm{exp}\left(i\hat{H}\left(B_{\mathrm{LF}}\right)\tau \right).$$

The $B_{\mathrm{LF}}\to B_{\mathrm{HF}}$ field jump is simulated numerically in the same way as the first field jump (the time interval is split into many small steps). Finally, knowing the density operator $\sigma_{\mathrm{fin}}$ at $t=t_{1}+\tau +t_{2}$ , we evaluate the NMR signals of the nuclei of interest as the expectation values of their z magnetization $\langle I_{iz}\rangle =\text{Tr}\left\{{\hat{I}}_{iz}\sigma_{\mathrm{fin}}\right\}$ .

The method used for modeling the experiments with decoupling at $B=B_{\mathrm{LF}}$ is different. After evaluating the density operator $\sigma (t=t_{1})$ , we trace out the proton degree of freedom and define the density operator of the carbon subsystem as $\sigma_{C}\left(t_{1}\right)={\text{Tr}}_{H}\left\{\sigma (t_{1})\right\}$ , with the argument that proton polarization is destroyed by decoupling. The partial trace procedure implies that when $\sigma_{ik,jl}$ is a proton–carbon density operator (in the notation of spin states, ij stand for the proton states and kl stand for the carbon-13 states), the elements of the carbon density operator are ${\left\{\sigma_{C}\right\}}_{k,l}=\sum_{i}\sigma_{ik,il}$ . One should note that proton two-spin operators may contain a zero-quantum component which would withstand proton decoupling. Consideration of the effects of such coherences is beyond the scope of this work; we expect this to only lead to small perturbations of the observed behavior. Then, we introduce the following Hamiltonian of the carbon-13 subsystem:

12 $${\hat{H}}_{C}\left(B_{\mathrm{LF}}\right)=-\gamma_{C}B_{\mathrm{LF}}\sum_{i=1}^{3}\left(1+\delta_{Ci}\right){\hat{I}}_{iz}+2\pi \sum_{i\neq k}J_{Cik}\left(\hat{I_{i}}\cdot {\hat{I}}_{k}\right).$$

Using this Hamiltonian, we evaluate the density operator of the ${}^{13}C$ spins at the end of the evolution period as follows:

13 $$\sigma_{C}\left(t_{1}+\tau \right)=\mathrm{exp}\left(-i{\hat{H}}_{C}\left(B_{\mathrm{LF}}\right)\tau \right)\sigma_{C}\left(t_{1}\right)\mathrm{exp}\left(i{\hat{H}}_{C}\left(B_{\mathrm{LF}}\right)\tau \right).$$

The final step in evaluating the ZQC evolution is introducing the carbon-13–proton density operator. This is done by multiplying $\sigma_{C}\left(t_{1}+\tau \right)$ and the density operator of nonpolarized protons (as decoupling removes any proton spin order). Hence, we determine the following:

14 $$\sigma \left(t_{1}+\tau \right)=\sigma_{C}\left(t_{1}+\tau \right)⊗\sigma_{H}^{\mathrm{dec}},\;\;\sigma_{H}^{\mathrm{dec}}=\frac{1}{2^{7}}\prod_{j=1}^{7}\hat{1},$$

where $\hat{1}$ is the identity operator. The final step of the calculation, the field jump $B_{\mathrm{LF}}\to B_{\mathrm{HF}}$ , is modeled in the same way as in the previous case.

Finally, we would like to comment on the B(t) dependence which was used in calculation. The distance dependence of the magnetic field B(z) is precisely known, but the precise z(t) is not known. We modeled this dependence assuming that motion goes with a constant speed (in experiments constant-speed motion is achieved after a 5–10 ms lag delay for acceleration). Nonideal agreement between theory and experiment can be attributed to the fact that the precise z(t) dependence is not known; our previous works (Pravdivtsev et al., 2013; Kiryutin et al., 2013) show that the knowledge of z(t) is required for modeling.

**Figure 5:**
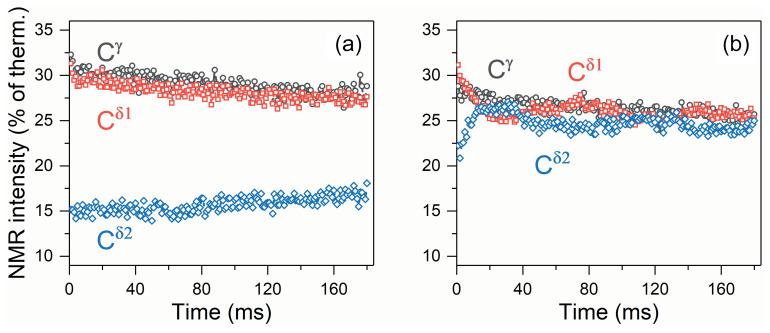
Observed τ dependence of the polarizations of carbon-13 nuclei, $C^{\gamma }$ , $C^{\delta 1}$ and $C^{\delta 2}$ , measured **(a)** without ${}^{1}H$ decoupling and **(b)** with ${}^{1}H$ decoupling. The NMR intensities are plotted in the percent of the intensities of the NMR signals in the 150.9 MHz ${}^{13}C$ spectra (i.e., at 14.1 T) at thermal equilibrium.

## 4 Results and discussion

The experimental τ dependences of the measured spin polarization are shown in Fig. 5. One can see that without decoupling no coherent behavior is found; polarization simply decays due to relaxation, and no coherent oscillations are visible (Fig. 5a). In the presence of proton decoupling the situation is drastically different. Coherent oscillations are clearly observed, which mediate the polarization exchange between the $C^{\delta 1}$ and $C^{\delta 2}$ nuclei. We attribute such polarization exchange to the ZQC, which is generated by passage through the LAC. The coherence gives rise to exchange in the populations of the two states which experience the LAC. These levels are correlated with the |ααβ〉 and |αβα〉 high-field states. Hence, polarization transfer gives rise to population exchange in the states |ααβ〉 (initially overpopulated state) and |αβα〉 (initially underpopulated state). As a result, the state of the first spin, $C^{\gamma }$ , does not change, but the other two spins, $C^{\delta 1}$ and $C^{\delta 2}$ , exchange polarization. With the available speed and range of the field cycling, other coherences are not excited, i.e., nonadiabatic variation of the Hamiltonian is achieved only for the pairs of levels that have the LAC in between $B_{\mathrm{LF}}$ and $B_{\mathrm{HF}}$ (i.e., only the LAC shown in Fig. 4 contributes to spin mixing). The $C^{\gamma }$ spin never shows any oscillatory polarization transfer, which is an indication that the specific LAC is responsible for the observed effect. In conclusion, a zero-quantum coherence of the two carbon-13 nuclei, $C^{\delta 1}$ and $C^{\delta 2}$ , is excited by fast magnetic field jump between 14.1 and 0.33 T.

The oscillatory behavior does not show up in the absence of proton decoupling. There are two reasons for that. First, the multiple proton–carbon couplings give rise to a set of ZQC frequencies instead of a unique frequency in the presence of decoupling. Second, and more importantly, proton–carbon-13 couplings prevent the carbon subsystem from reaching the strong coupling regime. Thus, the amplitude of coherent evolutions is drastically reduced (see Eq. 7) and becomes negligible (Fig. 5a). As a result, in experiments without decoupling, the ZQC decays because of inhomogeneous broadening of the ZQC evolution frequency, i.e., relaxation. We would like to stress that the ZQC of interest is excited by the field jump, which is identical for experiments with and without proton decoupling at the low field. However, the ZQC does not reveal itself and does not give rise to efficient polarization transfer in the experiment without decoupling.

**Figure 6:**
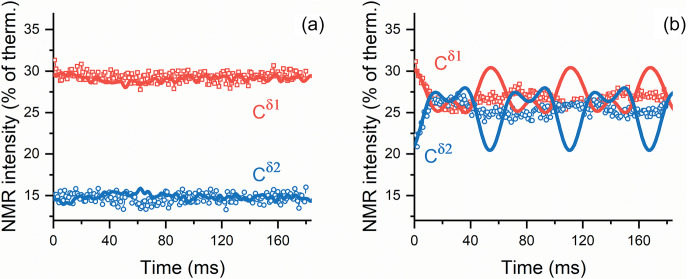
Calculated τ dependence of polarization (lines) overlaid with the observed time traces (points) obtained **(a)** without ${}^{1}H$ decoupling and **(b)** with ${}^{1}H$ decoupling. The slowly relaxing background (compare with the data shown in Fig. 5) has been subtracted from the time traces to enable comparison between theory and simulations. Observed NMR intensities are normalized to intensities in 150.9 MHz (14.1 T) ${}^{13}C$ spectra at thermal equilibrium. We use the subtraction procedure because relaxation effects were not taken into account in the calculation; consequently, we are unable to consider polarization decay due to relaxation at $B_{\mathrm{LF}}$ and during the field variation. To enable a comparison of the experiment and calculation results, the amplitude of oscillations in polarization transfer traces were scaled with the same factor; then, the starting polarization values were adjusted individually to give best agreement with the experimental data.

These considerations are confirmed by theoretical modeling (Fig. 6). In the presence of carbon–proton couplings, coherent oscillations are hardly observed; only fast oscillations of a very small amplitude can be seen in the simulated curves. By contrast, in the absence of the proton–carbon-13 couplings, i.e., when decoupling is used, coherent evolutions become manifest with slower oscillations of larger amplitude. The results of numerical modeling are in good agreement with the experimental data. As relaxation effects are not taken into account in simulations, to ease comparison we subtracted the slowly relaxing background from the experimental time traces. In addition, we rescaled all calculated traces with the same factor; then, the starting polarization values were adjusted individually to achieve the best agreement with the experimental data. Such a data treatment becomes necessary because relaxation is active not only during spin mixing at the $B_{\mathrm{LF}}$ field but also during the field jumps. The agreement between the experimental data and simulation in Fig. 6 is not ideal, possibly because some small long-range scalar couplings are not included in the simulation, but most likely because the field switching profile is not known exactly; previous studies of the spin dynamics in field-cycling NMR experiments (Pravdivtsev et al., 2013; Kiryutin et al., 2013) suggest that using the precise B(t) profile is crucial for simulating coherent polarization transfer phenomena.

The absence of a strong coupling regime, in spite of scalar-coupling constants larger than the difference in Larmor frequencies, is somewhat counterintuitive but clearly explained when taking into account the effect of large heteronuclear scalar couplings (Eqs. 2–4). In the present case, the effect is even more pronounced since the two δ carbon-13 nuclei of leucine are coupled to no less than three protons each, further splitting resonance frequencies in the absence of proton decoupling. A conventional way to present the weak coupling regime consists of stating that the part of the scalar-coupling Hamiltonian (Eq. 1) that is proportional to a zero-quantum product operator is nonsecular in the frame of the Zeeman interactions of the two coupled spins, which is true if the scalar-coupling constant is much smaller than the difference in Larmor frequencies of the two spins. Here, the weak coupling regime is extended because this zero-quantum part can be considered nonsecular in the interaction frame of the heteronuclear scalar couplings (note that the perturbative treatment is allowed to the extent that the heteronuclear coupling constants are much larger than the homonuclear coupling).

A particular consequence of the observation we report here can be relevant for experiments in which the strong scalar coupling regime is created by radio frequency irradiation, i.e., isotropic mixing for TOCSY (Braunschweiler and Ernst, 1983). We have recently introduced a two-field TOCSY experiment in which isotropic mixing is carried out at 0.33 T and chemical shift evolutions occur at the high field (Kadeřávek et al., 2017), which makes broadband carbon-13 TOCSY straightforward. This study included a control experiment where no radio frequency pulses were applied at the low field (see Fig. 3b in Kadeřávek et al., 2017). Intuitively, one would have expected cross peaks to be observed for carbon-13 nuclei in strongly coupled networks at 0.33 T. Some cross peaks could indeed be observed within the aliphatic carbon region of leucine and in the aromatic ring of phenylalanine. The current investigation suggests that strong scalar couplings between carbon-13 nuclei are less prevalent than expected at 0.33 T. The observed cross peaks were possibly due to cross relaxation and not necessarily coherent evolution under strong scalar couplings. Conventional TOCSY experiments might also be altered by the effect of large heteronuclear scalar couplings. In this case, isotropic mixing sequences have been optimized on isolated pairs of two coupled spins (Kadkhodaie et al., 1991), excluding the effects of scalar couplings to heteronuclei or as heteronuclear decoupling sequences that happen to be efficient at isotropic mixing (Rucker and Shaka, 1989; Shaka et al., 1988). Although isotropic mixing sequences decouple heteronuclear scalar couplings, optimizing simultaneously for homo- and heteronuclear scalar coupling operators may improve homonuclear coherence transfers. Such effects of couplings to heteronuclei are of relevance for abundant nuclei such as protons or ${}^{13}C$ spins in uniformly ${}^{13}C$ -labeled molecules.

## 5 Conclusions

In this work, we present a study of coherent polarization transfer in a system of (strongly) coupled ${}^{13}C$ nuclei. Spin coherences are zero-quantum coherences, which are generated by a fast nonadiabatic magnetic field jump. Such coherences are excited most efficiently when the system goes through a LAC during the field switch. Here we indeed pass through a LAC in a system of three coupled ${}^{13}C$ spins and investigate the spin dynamics at low fields, where strong couplings of the carbon spins are expected.

We can clearly demonstrate that the polarization transfer in the carbon-13 spin subsystem is strongly affected by spin–spin interactions with the protons in the molecule. In this situation, the role of these interactions can be determined by comparing the experiments with and without proton decoupling at low fields. When decoupling is used, we observe coherent polarization exchange between two of the three carbons; such behavior is typical when the spin coherences are excited upon nonadiabatic passage through a specific LAC. In the absence of decoupling, i.e., when heteronuclear interactions are present, we cannot observe such behavior; polarization transfer is very inefficient and coherent phenomena are not found. We attribute this to the fact that relatively strong proton–carbon couplings (i) drive the carbon system away from the strong coupling condition and (ii) give rise to a set of evolution frequencies instead of a unique ZQC frequency. These considerations are supported by an analytical model of a three-spin system and numerical simulations in a multispin system.

Our results are of importance for analyzing polarization transfer phenomena at low magnetic fields and for interpreting NMR data obtained under apparently strong coupling conditions. Under such conditions, heteronuclear spin–spin interactions might disturb strong coupling of homonuclei and substantially alter spin dynamics. Similar effects also often arise in dynamic nuclear polarization where the difference in the electron–nuclear couplings for nuclei located at different distances from the electron hampers nuclear spin diffusion, giving rise to the spin diffusion barrier around the electron spin (Ramanathan, 2008).

## Supplement

10.5194/mr-1-237-2020-supplement The supplement related to this article is available online at: https://doi.org/10.5194/mr-1-237-2020-supplement.

## Code availability

Scripts used to run the numerical modeling of the polarization transfer in a carbon-13 subsystem of leucine in the presence of and in the absence of proton decoupling are provided in the Supplement.
