# Supplementary material for: Surprising absence of strong homonuclear coupling at low magnetic field explored by two-field nuclear magnetic resonance spectroscopy
Source: Magn Reson (Gott). 2020 Oct 14;1(2):237–46. doi: 10.5194/mr-1-237-2020 (PMC10726027; doi:10.5194/mr-1-237-2020)
Supplement: The supplement related to this article is available online at: https://doi.org/10.5194/mr-1-237-2020-supplement. [file mr-1-237-supplement.pdf]

Supplement of Magn. Reson., 1, 237–246, 2020
https://doi.org/10.5194/mr-1-237-2020-supplement
© Author(s) 2020. This work is distributed under
the Creative Commons Attribution 4.0 License.

Open Access
MAGNETIC RESONANCE

Supplement of

Surprising absence of strong homonuclear coupling at low magnetic field explored by two-field nuclear magnetic resonance spectroscopy

Ivan V. Zhukov et al.

Correspondence to: Konstantin L. Ivanov (ivanov@tomo.nsc.ru) and Fabien Ferrage (fabien.ferrage@ens.psl.eu)

The copyright of individual parts of the supplement might differ from the CC BY 4.0 License.

Content

Matlab-script used to model evolution of carbon-13 nuclei of the carbon-13 labeled leucine isopropyl group at low field, without ^{1}H decoupling - evolution_low_field_10_spins.m 2

Matlab-script used to model evolution of carbon-13 nuclei of the carbon-13 labeled leucine isopropyl group at low field, with ^{1}H decoupling - evolution_low_field_10_spins_proton_decoupling.m................ 5

Matlab-script used to calculate propagators *QfsUp* and *QfsDown* describing evolution of full 10-spin density matrix during sample transfer, from high field to low field and back - Propagator_10_spins.m ... 8

All simulations are done using Matlab R2014a (MATLAB 2014a, The MathWorks, Natick, 2014).

evolution_low_field_10_spins.m

```
% I.Zhukov 28.03.2019

% Warning! If you wish to use another magnetic field switching profile (change transfer time,
% acceleration, etc.), you should first calculate matrix of corresponding propogator using
% script "Propogator_10_spins.m".
clear all
'evolution_low_field_10_spins'
tic % clock start
filename='1,1,-1 1H_eq CS'; % User defined name of file

%******** propagator filenames ****************************
% left side propogator 14.1T -> 0.33T secular J_CH Paris linear profile
filename_QfsUp='QfsUp_10s_107ms_108_fps_CS.mat';
% left side propogator 0.33T -> 14.1T secular J_CH Paris linear profile
filename_QfsDown='QfsDown_10s_94ms_95_fps_CS.mat';
%*********************************************************************

%******** definition of initial spin order ****************
    p1=1;       % initial Cg polarization
    p2=1;       % initial Cd1 polarization
    p3=-1;       % initial Cd2 polarization
    enh_13C=1; % 13C enhancement factor acquired during preparation period


    pHg=1;      % initial Hg polarization
    pHd1=1;     % initial Hd1 polarization
    pHd2=1;     % initial Hd2 polarization
    pH_end=1;   % 1H polarization after decoupling at low field
%***********************************************************


B_low=330.8; % B_low [mT]
ev_time=0.2; % maximum delay at low field [s]
e_npoints = 200; % number of points to calculate evolution of density matrix at low field
dt_e = ev_time/e_npoints;

%******** parameters of spin system *************%
TypeNucH=42.5775e6; % gamma_1H [Hz/T]
TypeNucC=10.7084e6; % gamma_13C [Hz/T]
ChShC=[24.1 21.95 20.8]; %Measured 600MHz Cg Cd1 Cd2 chemical shifts
ChShH=[1.63 0.888 0.876]; %Measured 600MHz Hg Hd1 Hd2 chemical shifts
%13C-13C coupling constants
Jgd1=35.0;
Jgd2=35.4;
%13C-1H direct coupling constants
Jd1=124.8;
Jd2=124.8;
Jg=127.4;
%************************************************%

%******** spin operators of spin=1/2 ************%
I = complex(0,1);
U2=eye(2); % unity matrix 2x2
x=[0.0 0.5;0.5 0.0]; % Sx
y=[0.0 -0.5*I;0.5*I 0.0]; %Sy
z=[0.5 0.0;0.0 -0.5]; %Sz
%************************************************%

%************** definition of I_x,y,z spin operators for the spin system ****************%
ES10=eye(1024); % unity matrix 1024x1024

ICgx=kron(kron(kron(kron(kron(kron(kron(kron(kron(x,U2),U2),U2),U2),U2),U2),U2),U2),U2);
ICgy=kron(kron(kron(kron(kron(kron(kron(kron(kron(y,U2),U2),U2),U2),U2),U2),U2),U2),U2);
ICgz=kron(kron(kron(kron(kron(kron(kron(kron(kron(z,U2),U2),U2),U2),U2),U2),U2),U2),U2);


ICd1x=kron(kron(kron(kron(kron(kron(kron(kron(kron(U2,x),U2),U2),U2),U2),U2),U2),U2),U2);
ICd1y=kron(kron(kron(kron(kron(kron(kron(kron(kron(U2,y),U2),U2),U2),U2),U2),U2),U2),U2);
ICd1z=kron(kron(kron(kron(kron(kron(kron(kron(kron(U2,z),U2),U2),U2),U2),U2),U2),U2),U2);
```

```
ICd2x=kron(kron(kron(kron(kron(kron(kron(kron(kron(U2,U2),x),U2),U2),U2),U2),U2),U2),U2);
ICd2y=kron(kron(kron(kron(kron(kron(kron(kron(kron(U2,U2),y),U2),U2),U2),U2),U2),U2),U2);
ICd2z=kron(kron(kron(kron(kron(kron(kron(kron(kron(U2,U2),z),U2),U2),U2),U2),U2),U2),U2);

IHgz=kron(kron(kron(kron(kron(kron(kron(kron(kron(U2,U2),U2),z),U2),U2),U2),U2),U2),U2);

IHd11z=kron(kron(kron(kron(kron(kron(kron(kron(kron(U2,U2),U2),U2),z),U2),U2),U2),U2),U2);
IHd12z=kron(kron(kron(kron(kron(kron(kron(kron(kron(U2,U2),U2),U2),U2),z),U2),U2),U2),U2);
IHd13z=kron(kron(kron(kron(kron(kron(kron(kron(kron(U2,U2),U2),U2),U2),U2),z),U2),U2),U2);

IHd21z=kron(kron(kron(kron(kron(kron(kron(kron(kron(U2,U2),U2),U2),U2),U2),U2),z),U2),U2);
IHd22z=kron(kron(kron(kron(kron(kron(kron(kron(kron(U2,U2),U2),U2),U2),U2),U2),U2),z),U2);
IHd23z=kron(kron(kron(kron(kron(kron(kron(kron(kron(U2,U2),U2),U2),U2),U2),U2),U2),U2),z);
%*****************************************************************************************%

%******** definition of initial density matrix at high field ***********%
rho_start=ES10+enh_13C*(p1*ICgz+p2*ICd1z+p3*ICd2z)+TypeNucH/TypeNucC...
             *(pHg*IHgz+pHd1*(IHd11z+IHd12z+IHd13z)+pHd2*(IHd21z+IHd22z+IHd23z));
rho_start=rho_start/trace(rho_start);
%***********************************************************************%

%******** evolution of density matrix during B_high -> B_low field switching ***********%
% loading of 14.1 T -> 0.33 T propagator
var1_l = load(filename_QfsUp);
U1_l=var1_l.QfsUp;
% loading of 0.33 T -> 14.1 T propagator
var2_l = load(filename_QfsDown);
U2_l=var2_l.QfsDown;
% the definition of 10-spin system density matrix in the beginning of evolution period
rho_ev=U1_l*rho_start/U1_l;
clear var1_l var2_l
%***************************************************************************************%

%******** definition of low-field Hamiltonian ********************%
% J-couplings
ICg_ICd1=ICgx*ICd1x+ICgy*ICd1y+ICgz*ICd1z;
ICg_ICd2=ICgx*ICd2x+ICgy*ICd2y+ICgz*ICd2z;
ICg_IHg=ICgz*IHgz;
ICd1_IHd1=ICd1z*IHd11z+ICd1z*IHd12z+ICd1z*IHd13z;
ICd2_IHd2=ICd2z*IHd21z+ICd2z*IHd22z+ICd1z*IHd23z;

Jij=Jgd1*ICg_ICd1+Jgd2*ICg_ICd2+Jg*ICg_IHg+Jd1*ICd1_IHd1+Jd2*ICd2_IHd2;
% Zeeman interaction
Zeem_C_low=(-(ChShC(1)*1e-6)*ICgz...
            -(ChShC(2)*1e-6)*ICd1z...
            -(ChShC(3)*1e-6)*ICd2z)*TypeNucC*B_low*0.001;
Zeem_H_low=(-(1-TypeNucC/TypeNucH+ChShH(1)*1e-6)*IHgz...
         -(1-TypeNucC/TypeNucH+ChShH(2)*1e-6)*(IHd11z+IHd12z+IHd13z)...
         -(1-TypeNucC/TypeNucH+ChShH(3)*1e-6)*(IHd21z+IHd22z+IHd23z))...
             *TypeNucH*B_low*0.001;

Ham_low=Zeem_C_low+Zeem_H_low+Jij;  % total Hamiltonian = Zeeman + Scalar couplings
%*****************************************************************%

output = zeros(3, e_npoints);
time_list = zeros(1, e_npoints);
Ul=expm(-2*pi*I*Ham_low*dt_e);

%%%%%%%%%%%%%%%%%%%%%% MAIN CYCLE %%%%%%%%%%%%%%%%%%%%%%%%%%%

%******** evolution of spin system at low field ********************%
for iT=1:e_npoints
    iT
    rho_ev=Ul*rho_ev/Ul;
    %******** calculation of evolution during field switching 0.33 T -> 14.1 T ***%
    rho_fin=U2_l*rho_ev/U2_l;
```

```
%******** calculation of carbon-13 polarization ******************************%
    time_list(iT)=dt_e*iT;
    output(1,iT) = trace(rho_fin*ICgz);
    output(2,iT) = trace(rho_fin*ICd1z);
    output(3,iT) = trace(rho_fin*ICd2z);
end
%*****************************************************%

%%%%%%%%%%%%%%%%%%%%%%%%%%%%%%%%%%%%%%%%%%%%%%%%%%%%%%%%

%******************************* writing data to file **********************************%
DATA=[time_list.' output.'];
% concatenation of filename with calculation date and extension ".dat"
nytnavn = strcat(date, filename,'.dat');
save(nytnavn,'DATA','-ascii');
toc %end of clock
%***************************************************************************************%
```

evolution_low_field_10_spins_proton_decoupling.m

```
% I.Zhukov 28.03.2019

% Warning! If you wish to use another magnetic field switching profile (change transfer time,
% acceleration, etc.), you should first calculate matrix of corresponding propogator using
% script "Propogator_10_spins.m".
clear all
'evolution_low_field_10_spins_proton_decoupling'
tic % starts clock
filename=' gC 1,1,-1 1H decoupling constant speed'; % name of output file

%******************** propogator filename ***********************%
% left side propogator 14.1T -> 0.33T secular J_CH Paris linear
filename_QfsUp='QfsUp_10s_107ms_108_fps_CS.mat';
% left side propogator 0.33T -> 14.1T secular J_CH Paris linear
filename_QfsDown='QfsDown_10s_94ms_95_fps_CS.mat';
%****************************************************************%

%******** definition of initial spin order ****************%
    p1=1;       % initial Cg polarization
    p2=1;       % initial Cd1 polarization
    p3=-1;       % initial Cd2 polarization
    enh_13C=1; % 13C enhancement factor acquired during preparation period

    pHg=1;      % initial Hg polarization
    pHd1=1;     % initial Hd1 polarization
    pHd2=1;     % initial Hd2 polarization
%**********************************************************%


B_low=330.8; % B_low [mT]

ev_time=0.2; % maximum delay at low field [s]
e_npoints = 200; % number of points to calculate evolution of density matrix at low field
dt_e = ev_time/e_npoints;

%******** parameters of spin system *************%
TypeNucH=42.5775; % gamma_1H [MHz/T]
TypeNucC=10.7084; % gamma_13C [MHz/T]
ChShC=[24.1 21.95 20.8]; %Cg Cd1 Cd2
ChShH=[1.63 0.888 0.876]; %Hg Hd1 Hd2
%13C-13C coupling constants
Jgd1=35;
Jgd2=35.4;
Jd1d2=0;
%************************************************%

%******** spin operators of spin=1/2 ************%
I = complex(0,1);
U2=eye(2); % unity matrix 2x2
x=[0.0 0.5;0.5 0.0]; % Sx
y=[0.0 -0.5*I;0.5*I 0.0]; %Sy
z=[0.5 0.0;0.0 -0.5]; %Sz
%************************************************%

%* definition of I_x,y,z spin operators for the full proton and carbon-13 10-spin system

ES10=eye(1024); % unity matrix 1024x1024

Cgx=kron(kron(kron(kron(kron(kron(kron(kron(kron(x,U2),U2),U2),U2),U2),U2),U2),U2),U2);
ICgy=kron(kron(kron(kron(kron(kron(kron(kron(kron(y,U2),U2),U2),U2),U2),U2),U2),U2),U2);
ICgz=kron(kron(kron(kron(kron(kron(kron(kron(kron(z,U2),U2),U2),U2),U2),U2),U2),U2),U2);

ICd1x=kron(kron(kron(kron(kron(kron(kron(kron(kron(U2,x),U2),U2),U2),U2),U2),U2),U2),U2);
ICd1y=kron(kron(kron(kron(kron(kron(kron(kron(kron(U2,y),U2),U2),U2),U2),U2),U2),U2),U2);
ICd1z=kron(kron(kron(kron(kron(kron(kron(kron(kron(U2,z),U2),U2),U2),U2),U2),U2),U2),U2);
```

```
ICd2x=kron(kron(kron(kron(kron(kron(kron(kron(kron(U2,U2),x),U2),U2),U2),U2),U2),U2),U2);
ICd2y=kron(kron(kron(kron(kron(kron(kron(kron(kron(U2,U2),y),U2),U2),U2),U2),U2),U2),U2);
ICd2z=kron(kron(kron(kron(kron(kron(kron(kron(kron(U2,U2),z),U2),U2),U2),U2),U2),U2),U2);

IHgz=kron(kron(kron(kron(kron(kron(kron(kron(kron(U2,U2),U2),z),U2),U2),U2),U2),U2),U2);

IHd11z=kron(kron(kron(kron(kron(kron(kron(kron(kron(U2,U2),U2),U2),z),U2),U2),U2),U2),U2);
IHd12z=kron(kron(kron(kron(kron(kron(kron(kron(kron(U2,U2),U2),U2),U2),z),U2),U2),U2),U2);
IHd13z=kron(kron(kron(kron(kron(kron(kron(kron(kron(U2,U2),U2),U2),U2),U2),z),U2),U2),U2);

IHd21z=kron(kron(kron(kron(kron(kron(kron(kron(kron(U2,U2),U2),U2),U2),U2),U2),z),U2),U2);
IHd22z=kron(kron(kron(kron(kron(kron(kron(kron(kron(U2,U2),U2),U2),U2),U2),U2),U2),z),U2);
IHd23z=kron(kron(kron(kron(kron(kron(kron(kron(kron(U2,U2),U2),U2),U2),U2),U2),U2),U2),z);
%*****************************************************************************%

%******** definition of I_x,y,z spin operators for the carbon-13 3-spin system **********%
ICCgx=kron(kron(x,U2),U2);
ICCgy=kron(kron(y,U2),U2);
ICCgz=kron(kron(z,U2),U2);


ICCd1x=kron(kron(U2,x),U2);
ICCd1y=kron(kron(U2,y),U2);
ICCd1z=kron(kron(U2,z),U2);


ICCd2x=kron(kron(U2,U2),x);
ICCd2y=kron(kron(U2,U2),y);
ICCd2z=kron(kron(U2,U2),z);
%****************************************************************************%

%******** definition of initial density matrix at high field *************%
rho_start=ES10+enh_13C*(p1*ICgz+p2*ICd1z+p3*ICd2z) ...
      +TypeNucH/TypeNucC*(pHg*IHgz+pHd1*(IHd11z+IHd12z+IHd13z)+pHd2*(IHd21z+IHd22z+IHd23z));
rho_start=rho_start/trace(rho_start);
%*********************************************************************%

%******** evolution of density matrix during B_high -> B_low field switching ***********%
% loading of 14.1 T -> 0.33 T propagator
var1_l = load(filename_QfsUp);
U1_l=var1_l.QfsUp;
% loading of 0.33 T -> 14.1 T propagator
var2_l = load(filename_QfsDown);
U2_l=var2_l.QfsDown;
% the definition of 10-spin system density matrix in the beginning of evolution period
rho_ev=U1_l*rho_start/U1_l;
clear var1_l var2_l
%****************************************************************************%

%************** construction of 4 indices density matrix *****************%
rho_ev10_in = zeros(8,128,8,128);
for a1=1:1:8
    for a2=1:1:8
        for b1=1:1:128
            for b2=1:1:128
                II=128*(a1-1)+b1;
                JJ=128*(a2-1)+b2;
                rho_ev10_in(a1,b1,a2,b2)=rho_ev10(II,JJ);
            end
        end
    end
end
%************** calculation of trace over proton indices*****************%
rho_ev=zeros(8,8);
for a1=1:1:8
    for a2=1:1:8
        for b1=1:1:128
            rho_ev(a1,a2)=rho_ev(a1,a2)+rho_ev10_in(a1,b1,a2,b1);
        end
    end
end
%*********************************************************************%
```

```
%************************** definition of low-field Hamiltonian ***********************%
ICg_ICd1=ICCgx*ICCd1x+ICCgy*ICCd1y+ICCgz*ICCd1z;
ICg_ICd2=ICCgx*ICCd2x+ICCgy*ICCd2y+ICCgz*ICCd2z;
ICd1_ICd2=ICCd1x*ICCd2x+ICCd1y*ICCd2y+ICCd1z*ICCd2z;

Jij=Jgd1*ICg_ICd1+Jgd2*ICg_ICd2+Jd1d2*ICd1_ICd2;  %J-couplings

Zeem_C_low=(-(ChShC(1)*1e-6)*ICCgz...   % Zeeman interaction
                     -(ChShC(2)*1e-6)*ICCd1z...
                     -(ChShC(3)*1e-6)*ICCd2z)*TypeNucC*B_low*0.001;
Ham_low=Zeem_C_low+Jij;  % total Hamiltonian = Zeeman + J-couplings
%*************************************************************************************%

output = zeros(3, e_npoints);
time_list = zeros(1, e_npoints);
Ul=expm(-2*pi*I*Ham_low*dt_e);
ES7=kron(kron(kron(kron(kron(kron(U2,U2),U2),U2),U2),U2),U2);
ES7=ES7/trace(ES7);

%%%%%%%%%%%%%%%%%%%%%% MAIN CYCLE %%%%%%%%%%%%%%%%%%%%%%%%%%%%%

%********* evolution of spin system at low field ******************************%
for iT=1:e_npoints
    iT
    %************ evolution of the spin system at low field *****************%
    rho_ev=Ul*rho_ev/Ul;

    %*  calculation of evolution during field switching 0.33 T -> 14.1 T *%
    rho_C_ev=kron(rho_ev,ES7); % definition of 10-spin system after evolution at 0.33 T
    rho_fin=U2_l*rho_C_ev/U2_l;
    %* end of calculation of evolution during field switching 0.33 T -> 14.1 T *%

    %************** calculation of carbon-13 polarization *****************************%
    time_list(iT)=dt_e*iT;
    output(1,iT) = trace(rho_fin*ICgz);
    output(2,iT) = trace(rho_fin*ICd1z);
    output(3,iT) = trace(rho_fin*ICd2z);
end

%%%%%%%%%%%%%%%%%%%%%%%%%%%%%%%%%%%%%%%%%%%%%%%%%%%%%%%%%

%******************************** writing data to file **********************************%
DATA=[time_list.' output.'];
% concatenated filename with date of calculation and extension ".dat"
nytnavn = strcat(date,filename,'.dat');
save(nytnavn,'DATA','-ascii');
toc %end of clock
%*************************************************************************************%
```

Propagator_10_spins.m

```
clear all
tic % starts clock
% left hand side propagator 14.1 T -> 0.33 T filename
filename_QfsUp='QfsUp_10s_107ms_108_fps_CS.mat';
% left hand side propagator 0.33 T -> 14.1 T filename
filename_QfsDown='QfsDown_10s_94ms_95_fps_CS.mat';

B_high=14092.4; % B_high [mT]
B_low=330.8; % B_low [mT]
dt=0.001; % time increment for propagator calculation

%* magnetic field at corresponding positions of the sample during transfer 14.1 T -> 0.33 T *%
fields_UP=[14.0924  14.0924 14.09239    14.09239    14.09238    14.09238    14.09239
14.09241    14.09245    14.0925 14.09256    14.0926 14.09258    14.09244    14.0921 14.09133
14.08985    14.08737    14.08362    14.07813    14.07033    14.05963    14.04542    14.027
14.00364    13.97458    13.93909    13.89638    13.84567    13.78615    13.71698    13.63729
13.54618    13.44267    13.32571    13.1942 13.04692    12.88256    12.69959    12.4965
12.27172    12.0238 11.75138    11.45358    11.13045    10.78288    10.41338    10.02523
9.62313 9.21188 8.79613 8.38025 7.96782 7.56202 7.16523 6.77953 6.4064  6.0471  5.70238
5.37279 5.05857 4.75983 4.4764  4.20802 3.95435 3.71501 3.48949 3.27725 3.07775 2.89044
2.71472 2.54994 2.39552 2.25091 2.11557 1.98892 1.87043 1.75959 1.65594 1.55905 1.46847
1.38375 1.30453 1.23049 1.16133 1.09669 1.03629 0.97992 0.92739 0.87851 0.83315 0.79128
0.75286 0.71784 0.68631 0.65862 0.63369 0.60756 0.57289 0.51563 0.43522 0.36699 0.33715
0.33085 0.33026 0.33071 0.3307  0.3308];
%*****************************************************************************************%

%* magnetic field at corresponding positions of the sample during transfer 14.1 T -> 0.33 T *%
fields_DOWN=[0.3308 0.33077 0.33072 0.33067 0.33069 0.33075 0.33069 0.33033 0.3304  0.33333
0.35538 0.4253  0.51726 0.57991 0.61618 0.64506 0.67494 0.7097  0.74916 0.79309 0.84155
0.89459 0.95231 1.01493 1.08276 1.15609 1.23533 1.32099 1.41356 1.51351 1.62145 1.73806 1.864
1.99996 2.1467  2.30503 2.47581 2.65985 2.85801 3.07124 3.30046 3.54648 3.81013 4.09221 4.3934
4.71424 5.05513 5.41634 5.79771 6.19877 6.6186  7.0557  7.50795 7.97243 8.44554 8.92246
9.39755 9.864   10.31469    10.74242    11.14176    11.509  11.84292    12.14378    12.41327
12.65355    12.8669 13.05564    13.22203    13.36805    13.49546    13.6059 13.70088
13.7818 13.84997    13.90665    13.95305    13.99036    14.01972    14.04225    14.05908
14.07124    14.07968    14.08527    14.08884    14.09095    14.09201    14.09243    14.09258
14.09259    14.09253    14.09246    14.09241    14.09238    14.0924];
%*****************************************************************************************%

dTUp_nps=108;
dTDown_nps=95;

%parameters of spin system
TypeNucH=42.5775e6; % gamma_1H [Hz/T]
TypeNucC=10.7084e6; % gamma_13C [Hz/T]
ChShC=[24.1 21.95 20.8]; %Cg Cd1 Cd2 400 MHz
ChShH=[1.63 0.888 0.876]; %Hg Hd1 Hd2
%13C-13C coupling constants
Jgd1=35;
Jgd2=35.4;
Jd1d2=0;
%13C-1H direct coupling constants
Jd1=124.8;
Jd2=124.8;
Jg=127.4;
%1H-1H coupling comstants
JHgd1=6.25;
JHgd2=6.25;

% spin operators of spin=1/2
I = complex(0,1);
U2=eye(2); % unity matrix 2x2
x=[0.0 0.5;0.5 0.0]; % Sx
y=[0.0 -0.5*I;0.5*I 0.0]; %Sy
z=[0.5 0.0;0.0 -0.5]; %Sz
```

```
%* definition of I_x,y,z spin operators for the full proton and carbon-13 10-spin system
ICgx=kron(kron(kron(kron(kron(kron(kron(kron(kron(x,U2),U2),U2),U2),U2),U2),U2),U2),U2);
ICgy=kron(kron(kron(kron(kron(kron(kron(kron(kron(y,U2),U2),U2),U2),U2),U2),U2),U2),U2);
ICgz=kron(kron(kron(kron(kron(kron(kron(kron(kron(z,U2),U2),U2),U2),U2),U2),U2),U2),U2);

ICd1x=kron(kron(kron(kron(kron(kron(kron(kron(kron(U2,x),U2),U2),U2),U2),U2),U2),U2),U2);
ICd1y=kron(kron(kron(kron(kron(kron(kron(kron(kron(U2,y),U2),U2),U2),U2),U2),U2),U2),U2);
ICd1z=kron(kron(kron(kron(kron(kron(kron(kron(kron(U2,z),U2),U2),U2),U2),U2),U2),U2),U2);

ICd2x=kron(kron(kron(kron(kron(kron(kron(kron(kron(U2,U2),x),U2),U2),U2),U2),U2),U2),U2);
ICd2y=kron(kron(kron(kron(kron(kron(kron(kron(kron(U2,U2),y),U2),U2),U2),U2),U2),U2),U2);
ICd2z=kron(kron(kron(kron(kron(kron(kron(kron(kron(U2,U2),z),U2),U2),U2),U2),U2),U2),U2);

IHgx=kron(kron(kron(kron(kron(kron(kron(kron(kron(U2,U2),U2),x),U2),U2),U2),U2),U2),U2);
IHgy=kron(kron(kron(kron(kron(kron(kron(kron(kron(U2,U2),U2),y),U2),U2),U2),U2),U2),U2);
IHgz=kron(kron(kron(kron(kron(kron(kron(kron(kron(U2,U2),U2),z),U2),U2),U2),U2),U2),U2);

IHd11x=kron(kron(kron(kron(kron(kron(kron(kron(kron(U2,U2),U2),U2),x),U2),U2),U2),U2),U2);
IHd11y=kron(kron(kron(kron(kron(kron(kron(kron(kron(U2,U2),U2),U2),y),U2),U2),U2),U2),U2);
IHd11z=kron(kron(kron(kron(kron(kron(kron(kron(kron(U2,U2),U2),U2),z),U2),U2),U2),U2),U2);

IHd12x=kron(kron(kron(kron(kron(kron(kron(kron(kron(U2,U2),U2),U2),U2),x),U2),U2),U2),U2);
IHd12y=kron(kron(kron(kron(kron(kron(kron(kron(kron(U2,U2),U2),U2),U2),y),U2),U2),U2),U2);
IHd12z=kron(kron(kron(kron(kron(kron(kron(kron(kron(U2,U2),U2),U2),U2),z),U2),U2),U2),U2);

IHd13x=kron(kron(kron(kron(kron(kron(kron(kron(kron(U2,U2),U2),U2),U2),U2),x),U2),U2),U2);
IHd13y=kron(kron(kron(kron(kron(kron(kron(kron(kron(U2,U2),U2),U2),U2),U2),y),U2),U2),U2);
IHd13z=kron(kron(kron(kron(kron(kron(kron(kron(kron(U2,U2),U2),U2),U2),U2),z),U2),U2),U2);

IHd21x=kron(kron(kron(kron(kron(kron(kron(kron(kron(U2,U2),U2),U2),U2),U2),U2),x),U2),U2);
IHd21y=kron(kron(kron(kron(kron(kron(kron(kron(kron(U2,U2),U2),U2),U2),U2),U2),y),U2),U2);
IHd21z=kron(kron(kron(kron(kron(kron(kron(kron(kron(U2,U2),U2),U2),U2),U2),U2),z),U2),U2);

IHd22x=kron(kron(kron(kron(kron(kron(kron(kron(kron(U2,U2),U2),U2),U2),U2),U2),U2),x),U2);
IHd22y=kron(kron(kron(kron(kron(kron(kron(kron(kron(U2,U2),U2),U2),U2),U2),U2),U2),y),U2);
IHd22z=kron(kron(kron(kron(kron(kron(kron(kron(kron(U2,U2),U2),U2),U2),U2),U2),U2),z),U2);

IHd23x=kron(kron(kron(kron(kron(kron(kron(kron(kron(U2,U2),U2),U2),U2),U2),U2),U2),U2),x);
IHd23y=kron(kron(kron(kron(kron(kron(kron(kron(kron(U2,U2),U2),U2),U2),U2),U2),U2),U2),y);
IHd23z=kron(kron(kron(kron(kron(kron(kron(kron(kron(U2,U2),U2),U2),U2),U2),U2),U2),U2),z);

 %*********************** Hamiltonian definition ***************************%
ICg_ICd1=ICgx*ICd1x+ICgy*ICd1y+ICgz*ICd1z;
ICg_ICd2=ICgx*ICd2x+ICgy*ICd2y+ICgz*ICd2z;
ICd1_ICd2=ICd1x*ICd2x+ICd1y*ICd2y+ICd1z*ICd2z;
ICg_IHg=ICgz*IHgz;
ICd1_IHd1=ICd1z*IHd11z+ICd1z*IHd12z+ICd1z*IHd13z;
ICd2_IHd2=+ICd2z*IHd21z+ICd2z*IHd22z+ICd1z*IHd23z;
IHg_IHd1=IHgz*IHd11z+IHgz*IHd12z+IHgz*IHd13z+...
            IHgx*IHd11x+IHgx*IHd12x+IHgx*IHd13x+...
            IHgy*IHd11y+IHgy*IHd12y+IHgy*IHd13y;
IHg_IHd2=IHgz*IHd21z+IHgz*IHd22z+IHgz*IHd23z+...
            IHgx*IHd21x+IHgx*IHd22x+IHgx*IHd23x+...
            IHgy*IHd21y+IHgy*IHd22y+IHgy*IHd23y;
%J-coupling part of Hamiltonian
Jij=Jgd1*ICg_ICd1+Jgd2*ICg_ICd2+Jd1d2*ICd1_ICd2+Jg*ICg_IHg+Jd1*ICd1_IHd1+Jd2*ICd2_IHd2+JHgd1*I
Hg_IHd1+JHgd2*IHg_IHd2;
Zeem_C_coef = (-(ChShC(1)*1e-6)*ICgz-(ChShC(2)*1e-6)*ICd1z-(ChShC(3)*1e-6)*ICd2z)*TypeNucC;
Zeem_H_coef = (-(1-TypeNucC/TypeNucH+ChShH(1)*1e-6)*IHgz...
               -(1-TypeNucC/TypeNucH+ChShH(2)*1e-6)*(IHd11z+IHd12z+IHd13z) ...
               -(1-TypeNucC/TypeNucH+ChShH(3)*1e-6)*(IHd21z+IHd22z+IHd23z))*TypeNucH;
% calculation of 14.1 T -> 0.33 T propagator
for iT=1:dTUp_nps;
    iT
    B00=fields_UP(iT);
    Zeem_C=Zeem_C_coef*B00;
    Zeem_H=Zeem_H_coef*B00;
```

```
    Ham=Zeem_C+Zeem_H+Jij;  % total Hamiltonian = Zeeman + J-coupling
    if(iT==1)
        QfsUp=expm(-2*pi*I*Ham*dt);
    else
        QfsUp=expm(-2*pi*I*Ham*dt)*QfsUp;
    end
end
% calculation of 0.33 T -> 14.1 T propagator
for iT=1:dTDown_nps;
    iT

    B00=fields_DOWN(iT);
    Zeem_C=Zeem_C_coef*B00;
    Zeem_H=Zeem_H_coef*B00;
    Ham=Zeem_C+Zeem_H+Jij;  % total Hamiltonian = Zeeman + J-coupling
    if(iT==1)
        QfsDown=expm(-2*pi*I*Ham*dt);
    else
        QfsDown=expm(-2*pi*I*Ham*dt)*QfsDown;
    end
end
%******************************** writing data to file ***********************************%
save(filename_QfsUp,'QfsUp','-mat');
save(filename_QfsDown,'QfsDown','-mat');
toc %end of clock
%*****************************************************************************************%
```
